# Prosthetic valve endocarditis due to highly beta-lactam-resistant *Streptococcus oralis*: a case report

**DOI:** 10.1099/acmi.0.000437

**Published:** 2022-11-03

**Authors:** Ippei Tanaka, Shinichiro Morioka, Arisa Honda, Ryoichi Miyazaki, Takeaki Wajima, Hidemasa Nakaminami, Tomoyuki Kato

**Affiliations:** ^1^​ Department of Pharmacy, Japanese Red Cross Musashino Hospital, Tokyo, Japan; ^2^​ Department of Clinical Microbiology, School of Pharmacy, Tokyo University of Pharmacy and Life Sciences, Tokyo, Japan; ^3^​ Disease Control and Prevention Center, National Center for Global Health and Medicine, Tokyo, Japan; ^4^​ Department of Cardiology, Japanese Red Cross Musashino Hospital, Tokyo, Japan; ^5^​ Department of Microbiology, Faculty of Pharmacy, Meijo University, Nagoya, Japan; ^6^​ Department of Infection Control, Japanese Red Cross Musashino Hospital, Tokyo, Japan

**Keywords:** drug resistance, endocarditis, heart valve prosthesis, *Streptococcus oralis*, vancomycin, viridans streptococci

## Abstract

There are limited reports of patients with prosthetic valve infective endocarditis (IE) or recurrent IE due to highly beta-lactam-resistant viridans group streptococci. We present a case in which a patient with native valve IE due to beta-lactam-susceptible *Streptococcus oralis* developed prosthetic valve IE due to highly beta-lactam-resistant *S. oralis *. A 79-year-old man with a history of native aortic valve IE caused by beta-lactam-susceptible *S. oralis* 21 months prior to admission and aortic valve replacement was admitted to our hospital with a 2-week history of general malaise and low-grade fever. Transesophageal echocardiography showed a 20 mm vegetation on the prosthetic aortic valve, and emergency cardiovascular surgery was performed on admission day 2. Three sets of blood cultures on admission were positive for highly beta-lactam-resistant *S. oralis *. Vancomycin and cefazolin were administered as initial treatment. After the surgery, the patient was given vancomycin and gentamicin for 2 weeks, followed by vancomycin for 4 weeks. He was relapse-free at the 6-month follow-up. For patients with native valve IE due to *S. oralis* who have undergone valve replacement more than 1 year earlier, given the possibility of methicillin-resistant *Staphylococcus aureus* as well as *S. oralis* resistance to beta-lactams, it may be advisable to start vancomycin as an initial treatment and continue it until the infecting micro-organism has been proven to be susceptible to beta-lactams.

## Introduction

*Streptococcus oralis *, a member of the viridans group streptococci (VGS), can be the causative organism of infective endocarditis (IE), and the emergence of penicillin-resistant strains is becoming increasingly recognized [1]. There are limited reports of patients with a history of native valve IE due to beta-lactam-susceptible VGS developing prosthetic valve IE or recurrent IE due to highly beta-lactam-resistant VGS with a penicillin minimum inhibitory concentration (MIC) ≥4 µg ml^−1^ and a ceftriaxone MIC ≥4 μg ml^−1^. The addition of vancomycin as an initial treatment for prosthetic valve IE is an option [2]. Herein, we describe a case in which a patient with native valve IE due to beta-lactam-susceptible *S. oralis* who had undergone prosthetic valve replacement more than 1 year earlier developed prosthetic valve IE due to highly beta-lactam-resistant *S. oralis *, which was successfully treated with the addition of vancomycin as an initial treatment.

## Case report

A 79-year-old man with no history of antimicrobial exposure in the 30 days prior to admission, immunosuppressive drug use, or intravenous drug use was admitted to our hospital with a 2-week history of general malaise and low-grade fever. The patient had a history of native aortic valve IE caused by beta-lactam-susceptible *S. oralis* (Table 1) 21 months prior to admission. He received ampicillin for 4 weeks after the blood cultures were negative and underwent aortic valve replacement. The patient had right femoral artificial head replacement for hip osteoarthritis 2 months prior to admission and received perioperative antimicrobial prophylaxis with cefazolin for 3 days.

**Table 1.** Minimum inhibitory concentrations of each antimicrobial agent and their interpretation against the *Streptococcus oralis* strains isolated from the patient

Antimicrobial agent	* S. oralis* in the first IE MIC (µg ml^−1^)	Interpretation*	* S. oralis* in PVE MIC (µg ml^−1^)	Interpretation*
Penicillin G	<0.03	S	4	R
Ampicillin	<0.06	S	>8	R
Cefotiam	<0.5	N	>4	N
Cefotaxime	<0.12	S	4	R
Ceftriaxone	<0.12	S	4	R
Cefepime	<0.5	S	>2	R
Meropenem	<0.12	N	2	N
Erythromycin	<0.12	S	>2	R
Azithromycin	<0.25	S	4	R
Clindamycin	<0.12	S	<0.12	S
Chloramphenicol	<4	S	<4	S
Vancomycin	1	S	0.5	S
Levofloxacin	1	S	1	S
Trimethoprim/sulfamethoxazole	<0.5	N	<0.5	N

^∗^Interpretation (susceptible, intermediate, or resistant) was based on the Clinical and Laboratory Standards Institute criteria (M100).

IE, infective endocarditis; MIC, minimum inhibitory concentration; N, no Clinical and Laboratory Standards Institute breakpoint; PVE, prosthetic valve endocarditis; R, resistant; S, susceptible; VGS, viridans group streptococci.

Vital signs on admission were as follows: temperature 37.9 °C, pulse 77 beats min^−1^, blood pressure 94/57 mmHg, respiratory rate 18 breaths min^−1^ and oxygen saturation 98 % in room air. Examination of the extremities revealed oedema of both lower extremities. Laboratory findings included a high C-reactive protein concentration (12.41 mg dl^−1^, reference range 0–0.14 mg dl^−1^) and white blood cell count (18 200 cells µl^−1^, reference 3300–8600 cells µl^−1^). A chest X-ray showed an increased cardiothoracic ratio compared to that 2 years prior to admission. We obtained three sets of blood cultures on admission that were positive for highly beta-lactam-resistant *S. oralis* (Table 1).

Transesophageal echocardiography revealed a 20 mm vegetation on the prosthetic aortic valve. A diagnosis of prosthetic aortic valve IE was made. The patient was transferred to a specialized facility for emergency cardiovascular surgery on admission day 2. He underwent aortic root replacement and coronary artery bypass grafting to avoid possible dispersal of the abscess on the same day. A ruptured aortic valve annulus with vegetation on the prosthetic aortic valve was observed during surgery; the culture was negative for *S. oralis *.

The patient was initially treated with a combination of vancomycin and cefazolin. On day 2, vancomycin and cefazolin were switched to levofloxacin at the specialized facility. On day 10, the patient was transferred to our hospital for postoperative care. Based on the antimicrobial susceptibility of *S. oralis,* levofloxacin was discontinued, and vancomycin and gentamicin were initiated. Repeated blood cultures were negative on day 10. After surgery, the patient received 2 weeks of vancomycin and gentamicin, followed by vancomycin for 4 weeks. At the end of 6 weeks of antibiotic therapy, the patient’s C-reactive protein concentration and white blood cell count decreased to 1.20 mg dl^−1^ and 6300 cells µl^−1^, respectively, and transthoracic echocardiography showed no evidence of valve dysfunction. During hospitalization, the patient visited the dental surgery department and was diagnosed with radicular periodontitis of the left maxilla and chronic marginal periodontitis. Extraction was performed for the maxillary apical periodontitis, and brushing care guidance was provided for the marginal periodontitis. The patient was relapse-free at the 6-month follow-up.

## Discussion

There are two important clinical and microbiological findings regarding IE. First, patients with a history of native valve IE due to beta-lactam-susceptible *S. oralis* can develop prosthetic valve IE due to highly beta-lactam-resistant *S. oralis. Streptococcus* is the most causative organism of IE in Japan (51.9%), while VGS accounts for 33.3 % of cases [3]. Shelburne *et al*. reported that beta-lactam-nonsusceptible VGS bloodstream infection is associated with prophylactic use of beta-lactams, treatment with beta-lactams within the past 30 days and nosocomial onset of infection [4]; however, none of these were present in our case. Four cases of VGS IE with a penicillin MIC ≥4 µg ml^−1^ and a ceftriaxone MIC ≥4 μg ml^−1^, as in our case, have been reported [5–7]; however, there is no report of recurrent IE due to VGS of the same species that became highly resistant to beta-lactam. To the best of our knowledge, this is the first reported case of highly beta-lactam-resistant VGS prosthetic valve IE in a patient with a history of beta-lactam-susceptible VGS native valve IE.

Second, the addition of vancomycin as an initial treatment option was effective for prosthetic valve IE in patients with native valve IE due to *S. oralis* who had undergone prosthetic valve replacement more than 1 year earlier. Guidelines recommend covering staphylococci, streptococci and enterococci as the initial choice of empirical treatment for prosthetic valve endocarditis more than 1 year post-operation[2, 8]. Guidelines recommend antibiotic therapy for staphylococci, VGS and enterococci in culture-negative prosthetic valve endocarditis with symptom onset more than 1 year after valve placement and list vancomycin and ceftriaxone as one initial treatment option [2]. The frequency of streptococcal IE in Japan is high [3]. Prabhu *et al*. reported that VGS detected in patients with IE are increasingly penicillin-resistant [9]. Teng *et al*. reported that high-level penicillin resistance (MIC ≥4.0 µg ml^−1^) was most frequent in *S. oralis* among VGS infections in Taiwan [1]. On the other hand, *Staphylococcus* is the most common causative organism of prosthetic valve IE globally (39.8%), and the risk of in-hospital death is increased in prosthetic valve IE and staphylococcal IE [10]. In this case, we used vancomycin and cefazolin as an initial treatment because of the history of *S. oralis* IE and prosthetic valve replacement more than 1 year earlier, oral invasion was presumed, and the involvement of antimicrobial resistance in *S. oralis* and methicillin-resistant *Staphylococcus aureus* could not be ruled out.

This study has some limitations. Both *S. oralis* detected in two IE episodes were identified by Rapid ID 32 Strep (bioMérieux, Lyon, France). The *S. oralis* isolate in the first IE was not preserved, and the genetic homology assessment and comparison of the mechanisms of acquisition of antimicrobial resistance within the patient could not be conducted. Therefore, it remains unclear whether the *S. oralis* isolate in the previous IE episode became resistant to beta-lactams, or different *S. oralis* isolates caused the two IE episodes. The patient’s previous antimicrobial exposure was 3 days of cefazolin 2 months prior to admission; however, it is unclear whether it contributed to the development of antimicrobial resistance in the isolate.

Patients with a history of native valve IE due to beta-lactam-susceptible *S. oralis* can develop prosthetic valve IE due to highly beta-lactam-resistant *S. oralis *. The addition of vancomycin as an initial treatment option was effective for prosthetic valve IE in patients with native valve IE due to *S. oralis* who had undergone prosthetic valve replacement more than 1 year earlier. For patients with native valve IE due to *S. oralis* who have undergone valve replacement more than 1 year earlier, given the possibility of methicillin-resistant *S. aureus* as well as *S. oralis* resistance to beta-lactams, it may be advisable to start vancomycin as an initial treatment and continue it until the infecting micro-organism has been proven to be susceptible to beta-lactams. Further studies are needed to clarify the epidemiological characteristics of IE patients with *S. oralis* showing high resistance to antimicrobial agents and the microbiological characteristics of the organism.
